# Severe Pediatric Asthma Therapy: Mepolizumab

**DOI:** 10.3389/fped.2022.920066

**Published:** 2022-07-01

**Authors:** Nicola Ullmann, Francesca Peri, Olivia Florio, Federica Porcaro, Elisa Profeti, Alessandro Onofri, Renato Cutrera

**Affiliations:** ^1^Pediatric Pulmonology & Respiratory Intermediate Care Unit, Sleep, and Long Term Ventilation Unit, Academic Department of Pediatrics (DPUO), Bambino Gesù Children's Hospital, IRCCS, Rome, Italy; ^2^Department of Medicine, Surgery, and Health Sciences, University of Trieste, Trieste, Italy; ^3^Respiratory Medicine Unit, University “Magna Graecia” of Catanzaro, Catanzaro, Italy

**Keywords:** asthma, biologics, treatment, children, adolescents, mepolizumab, anti-interleukin-5, antibodies

## Abstract

There is a growing need for advanced treatment in children with persistent and severe asthma symptoms. As a matter of fact, between 2 and 5% of asthmatic children experience repeated hospitalizations and poor quality of life despite optimized treatment with inhaled glucocorticoid plus a second controller. In this scenario, mepolizumab, a humanized monoclonal antibody, has proven to be effective in controlling eosinophil proliferation by targeting interleukin-5 (IL-5), a key mediator of eosinophil activation pathways. Mepolizumab is approved since 2015 for adults at a monthly dose of 100 mg subcutaneously and it has been approved for patients ≥ 6 years of age in 2019. Especially in children aged 6 to 11 years, mepolizumab showed a greater bioavailability, with comparable pharmacodynamics parameters as in the adult population. The recommended dose of 40 mg every 4 weeks for children aged 6 through 11 years, and 100 mg for patients ≥ 12 years provides appropriate concentration and proved similar therapeutic effects as in the adult study group. A marked reduction in eosinophil counts clinically reflects a significant improvement in asthma control as demonstrated by validated questionnaires, reduction of exacerbation rates, and the number of hospitalizations. Finally, mepolizumab provides a safety and tolerability profile similar to that observed in adults with adverse events mostly of mild or moderate severity. The most common adverse events were headache and injection-site reaction. In conclusion, mepolizumab can be considered a safe and targeted step-up therapy for severe asthma with an eosinophilic phenotype in children and adolescents.

## Introduction

Severe asthma is a highly heterogeneous disease due to the interaction between genetic predisposition, immune response, and environmental risk factors. This complex interaction influences the onset of symptoms, the clinical evolution, and the severity of illness (1, 2). Severe asthma affects about 0.5% of the general pediatric population and 4.5% of children with asthma (3), with a wide discrepancy between countries. Although the prevalence of severe asthma in childhood is low, it is associated with high morbidity, occasionally mortality, and significant healthcare burden (4). In addition, children with severe asthma report a poor quality of life due to persistent respiratory symptoms, recurrent life-threatening attacks, anxiety and emotional distress, missed school days, and side effects of oral corticosteroids (OCS) (5, 6).

A multidisciplinary assessment is required to exclude comorbidities and modifiable factors, confirm the diagnosis, and initiate the most appropriate treatment for these patients (7). The identification of different phenotypes and endotypes, based on cellular and molecular mechanisms and related biomarkers, may guide physicians in their therapeutic choice (8).

Recently, several innovative monoclonal antibody agents have been approved to target specific inflammatory type 2 mediators and improve uncontrolled severe asthma when added to basal treatment (9). However, it is necessary that pediatric pulmonologists are aware of the benefits and risks of these medications, as well as the practical implications of providing these options for their patients (10).

In this review, especially focused on mepolizumab, we present all current evidence on the indications, use, safety, and efficacy of this fully-humanized anti-interleukin-5 (IL-5) antibody for children with severe asthma.

## Search Methods

This review is based on the most significant results from an extensive PubMed search, conducted independently by three different researchers, using the search terms: “treatment in severe pediatric asthma,” “severe asthma,” “mepolizumab,” “anti-IL-5 therapy,” “anti–IL-5 antibody,” “children,” “adolescents,” and all different synonyms or word combinations. More relevance was given to clinical trials especially focused on mepolizumab treatment.

## Management of Severe Asthma in Pediatrics

### Definition of Severe Asthma

There is no uniformly accepted definition of severe asthma (11). Different options can be found in the scientific literature (12–15). However, international societies agree on assessing asthma severity based on the treatment level required to achieve and maintain adequate control.

According to the Global Initiative for Asthma (GINA) guidelines 2022, severe asthma isindicated by needing a high dose of ICS-LABA to maintain symptom control or uncontrolled asthma despite steps 4 and 5 of the care and management of contributory factors (12).

The European Respiratory Society and the American Thoracic Society (ERS/ATS) definitions of severe asthma require that patients have needed therapy with a high dose of ICS and a second controller, such as LABA, leukotriene modifier, or theophylline, for the previous year or have needed OCS for 50% or more of the year to prevent asthma from becoming uncontrolled or that cannot be controlled despite this therapy (13).

### Step-by-Step Diagnosis

Patients that need to be moved to GINA steps 4 and 5 care have to be referred to a tertiary center for a specialist evaluation first to confirm the diagnosis and subsequently to identify the best personalized treatment (7). In the beginning, it is very important to distinguish between “difficult to treat asthma,” due to modifiable factors, and true “therapy-resistant asthma,” which is unresponsive to standard medications (16, 17). Although a concomitance between these two conditions cannot always be excluded, uncontrolled asthma is frequently caused by treatment-related issues such as poor adherence to medication, inadequate or inappropriate inhalation technique (18, 19), and persistent exposure to adverse environmental factors (smoke, irritants, allergens, etc.) (20)or emotional factors.

Moreover, the presence of comorbidities, including allergic conditions (rhinitis, eczema, atopic dermatitis, food or drug allergy, etc), sinus disease, gastroesophageal reflux, obesity, obstructive sleep apnea, anxiety, and depression, may reduce the response to therapy (21–23).

Finally, the exclusion of asthma-mimicking conditions (24), such as tracheobronchomalacia, bronchopulmonary dysplasia, cystic fibrosis, primary ciliary dyskinesia, immunodeficiencies, obliterative bronchiolitis, pertussis, tuberculosis, vascular rings, and foreign body in toddlers (25) and vocal cord dysfunction, exercise-induced hyperventilation, and habitual cough in children and adolescents (26), is necessary.

Once all the following steps are carried out, that is, revising treatment issues, excluduing or treating comorbidities or other differential diagnoses, and confirming that it is severe asthma, it is possible to consider add-on medications, including biologics.

### Phenotype and Endotype-Guided Therapy

The introduction of biologics has revolutionized the care of severe asthma in adult and pediatric populations (27–29). Recent studies have suggested that physicians should characterize patients with severe asthma not only by their phenotype but also by their endotype before starting a biological treatment (30). This approach is in line with personalized medicine, which aims to achieve a better characterization of patients with the purpose to prescribe the most suitable treatment at an individual level (31).

The phenotype is the summation of clinical features while the endotype is determined by biomolecular mechanisms leading to the pathogenesis of the disease (32). To date, two endotypes of severe asthma have been described, based on the pathogenetic processes linked to airway inflammation: T2-low endotype and T2-high endotype (33).

The T2-low endotype is more frequent in adults and is characterized by the following: neutrophilic or paucigranulocytic inflammation, T-helper lymphocytes type 17, innate lymphoid type 3 cells, and IL-1, IL-8, IL-17, and IL-23 are the respective implied molecules (34, 35).

The T2-high endotype typically affects children and it is characterized by two specific characteristics: allergic sensitization and eosinophilia (1). Allergic asthma is the most common in childhood; it often presents an early onset, and it is associated with a family or personal history of allergic disease, a positive skin prick test, an elevated serum total IgE level, and increased fractional exhaled nitric oxide (FeNO) (36). Patients with eosinophilia and diffuse airway inflammation often are well responsive to corticosteroids (37, 38). However, a subgroup of T2-high asthma has poor control of symptoms despite corticosteroids, probably due to very high levels of type 2 inflammation. T2-high asthma constitutes 50% of mild to moderate asthma and probably a larger proportion of patients with more severe asthma (37).

In the T2-high endotype, T-helper lymphocytes type 2 signal the production of IL-4, IL-5, and IL-13, while innate lymphoid cells type 3 are activated by the epithelial alarmins TSLP, IL-33, and IL-25. Specifically, IL-4 and IL-13 promote B lymphocyte activation, inducing plasma cell formation, isotype switching to IgE, and their production. On the other hand, IL-5 induces chemoattraction, proliferation, and activation of eosinophils and also decreases their apoptosis (32).

Current research has suggested that these molecular pathways of severe asthma should guide the choice of the most adequate biologic treatment (39). The identification of biomarkers such as total IgE, peripheral eosinophil count, and FeNO, as a surrogate of mechanism generating and maintaining type 2 inflammation, helps design a personalized therapy for patients (30). In this scenario which is constantly and progressively oriented toward tailored treatments, mepolizumab might be the more indicated add-on target therapy for severe eosinophilic asthma.

## Mepolizumab as Target Therapy for Severe Eosinophilic Asthma

### License and Mechanism of Action

Mepolizumab (Nucala, GlaxoSmithKline) has been approved by the Food and Drug Administration (FDA) and the European Medicines Agency (EMA) in patients aged 12 years and older since November 2015 and was licensed in September 2019 in patients aged 6 years and older as an add-on maintenance therapy for severe eosinophilic asthma (40, 41). Mepolizumab is a murine humanized monoclonal antibody belonging to the IgG1 kappa subclass. It binds circulating IL-5 and prevents its interaction with IL-5 receptor alfa. Mepolizumab target (i.e., IL-5) is a 134-amino acid dimeric glycoprotein with a four-helix bundle motif, which consists of a 52-kDa homodimer. Mepolizumab specifically binds to the α-chain of IL-5 with an IC_50_ of <1 nM, a dissociation constant of 4.2 pM, and stoichiometry of 2.2, so that two IL-5 dimers are cross-linked by two molecules of mepolizumab. Therefore, through this mechanism of action, mepolizumab effectively inhibits IL-5 ligation to IL-5Rα (42). As mentioned above, IL-5 is fundamental for the recruitment, activation, and survival of eosinophils.

### Eligibility Criteria and Dosage

The eligibility criteria of treatment with mepolizumab for severe eosinophilic asthma in children and adolescents include blood eosinophilia > 0.15 × 10^9^/l in the absence of OCS treatment and > 0.3 × 10^9^/l in the previous year plus two or more asthma exacerbations requiring hospitalization or OCS treatment. In contrast to adults, among children with severe asthma, a smaller proportion of patients appear to have clear features of type 2 mucosal inflammation, including greater eosinophilia (43).

The recommended dosage of mepolizumab differs according to age: it is administered subcutaneously at a dose of 40 mg every 4 weeks in children aged 6–11 years and at a dose of 100 mg in children aged ≥ 12 years (40).

### Safety, Adverse Effects, and Contraindications

Safety evaluations of mepolizumab have raised no significant concerns so far (44–46). The most common adverse effects (incidence ≥ 5%) of mepolizumab included headache, injection site reaction (e.g., pain, erythema, swelling, itching, and burning sensation), back pain, and fatigue (40, 41, 47).

Mepolizumab is not to be used in patients with helminth infection, and whenever found, it needs to be treated before the biological drug can be prescribed. Mepolizumab is also contraindicated in the event of a hypersensitivity reaction (40, 41).

### Efficacy Data

Data about the efficacy of mepolizumab were collected from several promising clinical trials conducted on adults and children aged 12 years and older including Dose-Ranging Efficacy And Safety With Mepolizumab In Severe Asthma (DREAM) (44); Mepolizumab As Adjunctive Therapy In Patients With Severe Asthma (MENSA) (45); Steroid Reduction With Mepolizumab Study (SIRIUS) (46); and Mepolizumab Adjunctive Therapy In Subjects With Severe Eosinophilic Asthma (MUSCA) (48). Most recently, a multinational, non-randomized, open-label study was conducted on children aged 6 to 11 years by Gupta et al. (NCT02377427) (49, 50).

#### Asthma Control

The impact on health-related quality of life has been objectified through several validated questionnaires. The Asthma Control Questionnaires (ACQ-5 and ACQ-7) have been used in most studies (44–46, 49, 50)with a minimally clinically important difference (MCID) in total score (i.e., ≥ 0.5-point reduction from baseline) detected in half of the children in the first weeks (49) and up to 1 year of treatment (50). These results resemble those of adolescents and adults with MCID from baseline achieved from 42 to 59% of population studies (see Table 1). Best results were recorded around week 56, suggesting that treatment should not be discontinued before 1 year.

**Table 1:** Minimally clinically important difference (MCID) in children and adults/adolescents at end of treatment.

**MCID at end of treatment**				
**Study population**	**Study size** ***(N)***	**Study duration** ***(Wks)***	**Placebo** ***N (%)***	**Treatment group** ***N (%)***
*Children*				
Gupta et al. (50)	30	52	–	17/29 (59)
*Adults/adolescents*				
Pavord et al. (44) (DREAM)	616	52	77/153 (50)	222/452 (49)
Ortega et al. (45) (MENSA)	576	32	85/186 (46)	202/373 (54)
Bel et al. (46) (SIRIUS)	135	24	19/66 (29)	29/69 (42)
Chupp et al. (48) (MUSCA)	551	24	116/276 (42)	161/274 (59)

*N, absolute numbers; Wks, weeks*.

Another tool, the St. George's Respiratory Questionnaire (SGRQ), offers a wider perspective on treatment effectiveness and shows confirmed clinical improvement in all the domains (i.e., symptoms, activity, and impact) after 6 months of therapy compared to the placebo group (48). It is noteworthy that, despite a reduction in exacerbations risk in all the cited studies (see next paragraph), a clear subjective improvement has not always been reported as well (44).

#### Exacerbation Rate

Although the clinical experience with mepolizumab in pediatric asthma is often heterogeneous and with high rates of treatment discontinuation (51), this drug seems most effective in reducing exacerbations. A total of 10 children (28%) reported an exacerbation and more specifically only three patients had two exacerbations during the first 12 weeks of treatment (49). Another recently published case report showed improvement in asthma exacerbations in two children with eosinophilic nonallergic asthma treated with mepolizumab for 2 years (52). When compared to adolescents and adults, pediatric exacerbation rates were similar (53). As previously stated, a dissociation between clinical efficacy and symptoms has been noted since the first studies. We assume that the main effect of mepolizumab (i.e., reduction in blood eosinophils with modulation of eosinophilic airway inflammation) has more impact on exacerbations than asthma control perception. Thus, exacerbation risk and daily symptoms could be distinct features in patients with severe asthma.

#### Lung Function

Most studies detected a moderate effect on pulmonary function tests and only a few studies showed a significant improvement in forced expiratory volume during the first second (FEV_1_) compared to the placebo group (45, 48). As a matter of fact, a clear pattern of FEV_1_ changes from baseline has not been detected in children (49, 50). However, pediatric data are lacking and no studies including a placebo group are available. Baseline blood eosinophils diminish in 97% of patients treated with mepolizumab, but higher levels of blood eosinophils have not been defined as predictive factors for treatment response (54). The best response in terms of FEV_1_ improvement has been highlighted in patients with higher baseline sputum eosinophils (55). It can be hypothesized that patients with diffused airway eosinophilic inflammation present with worse baseline lung function. In this scenario, mepolizumab has a double rationale, targeting both local and systemic eosinophils.

Data are summarized in Table 2.

**Table 2:** Available studies on mepolizumab in adults, adolescents and children with summarized the main outcomes and results.

**Study characteristics**			**Outcomes**			
**Reference**	**Population** **(severe asthmatics)**	**Intervention**	**Asthma control**	**Exacerbation rate**	**Lung function (FEV_**1**_)**	**Main outcome**
Pavord et al. (44) (DREAM)	616 adults and adolescents, 12–74 years	Mepo IV every 4-week x 52 weeks at 75, 250, or 750 mg vs placebo.	AQLQ and ACQ scores *did not differ* significantly between groups.	Mepo *significantly reduced the rate of exacerbations* vs placebo: 75 mg reduced the number of severe exacerbations/patient/year by 48%, 250 mg by 39% and 750 mg by 52%.	FEV_1_ *did not differ* significantly from placebo.	A tenfold lower dose of Mepo was equally effective in reducing asthma exacerbations.
Ortega et al. (45) (MENSA)	576 adults and adolescents, 12–82 years	Mepo IV or SC every 4-weeks x 32 week at 75 or 100 mg vs placebo	ACQ score *showed a significant improvement* in the treatment groups.	Mepo *significantly reduced the rate of exacerbations* vs placebo: exacerbations necessitating an emergency department visit or hospitalization were reduced by 32% in the group receiving IV M. and by 61% in the group receiving SC M.	FEV_1_ *presented a significant increase* from baseline before/post bronchodilation in IV Mepo (+100/146 ml) and in the SC Mepo (+98/138 ml) vs placebo.	Mepo administered either IV or SC reduced asthma exacerbations.
Bel et al. (46) (SIRIUS)	135 adults and adolescents, 16–74 years	Mepo 100 mg SC every 4-weeks x 20-week vs placebo.	ACQ score *showed a significant improvement* of 0.52 points in the treatment group.	Mepo *significantly reduced the rate of exacerbations* of 32% vs placebo.	FEV_1_ *presented a non-significant trend* from baseline before and after bronchodilation in the Mepo group than in the placebo group.	Mepo had a significant glucocorticoid-sparing effect, with a median % reduction in the glucocorticoid dose of 50% in the Mepo group, with no reduction observed in the placebo group.
Chupp et al. (48) (MUSCA)	551 adults and adolescents, >12 years	Mepo 100 mg SC every 4-weeks x 24-week vs placebo.	SGRQ score *showed a significant improvement* from baseline.	Mepo *significantly reduced the rate of exacerbations* and emergency-room visits of 58% vs placebo. The annual rate of exacerbations requiring admission did not differ between groups.	Pre-bronchodilator FEV_1_ values *presented a significant improvement* vs placebo.	Mepo was associated with significant improvement in health-related quality of life in the treatment group vs placebo.
Gupta et al. (49)	36 children, 6–11 years	Mepo 40 mg (<40 kg) or 100 mg (≥40 kg) SC every 4-weeks for 12 weeks compared to severe asthmatics adults.	ACQ score *showed a trend* toward improved asthma control.	Ten (28%) children reported ≥1 on-treatment exacerbation with a total of 13 events. Four children (all in the 40 mg dose group) required an on-treatment hospitalization or ER visit.	There was *no clear pattern* of change in FEV_1_ from baseline.	Mepo SC 40 or 100 mg showed a positive clinical profile in children 6–11 years providing bodyweight-adjusted drug exposure within twofold of target adult exposure.

*AQLQ, Asthma Quality of Life Questionnaire; ACQ, Asthma Control Questionnaire; FEV_1_, forced expiratory volume during the first second; Mepo, Mepolizumab; SC, subcutaneous; SGRQ, St. George's Respiratory Questionnaire; IV, intravenous*.

### Recommendation on Discontinuation and Predictor Response Criteria

There is no validated recommendation on mepolizumab discontinuation, and GINA guidelines 2022 suggest an initial trial of at least 4 months (12). The National Institute for Health and Care Excellence (NICE) guidelines suggest reevaluating patients after 12 months and continuing the treatment if exacerbations have been reduced by ≥ 50% (56). However, several studies have reported that patients who discontinued mepolizumab showed an increase in Asthma Control Questionnaire-5 (ACQ-5) score, asthmatic attacks rate, and peripheral eosinophilia (57).

Currently, there are no standardized response criteria for mepolizumab. Blood eosinophil count and the increase in lung function are considered the main parameters of therapy response. Moreover, the improvement of quality of life and physical fitness and the reduction of exacerbations have also been reported as clinical predictor tools to evaluate treatment benefit (54, 58).

The most recent GINA guidelines published in 2022 suggested the following potential predictors of good response to anti-IL5 treatment: (1) higher blood eosinophils, (2) higher number of severe exacerbations in the previous year, (3) adult-onset asthma, (4) adult-onset asthma, (5) nasal polyposis, (6) maintenance oral corticosteroids, and (7) low lung function.

### Other Indications in Children or Adolescents

To date, mepolizumab is also approved for the treatment of pediatric patients aged ≥12 years with hypereosinophilic syndrome (HES) at a dose of 300 mg every 4 weeks. The recommended patient population includes those with ≥ 6 month duration of HES without an identifiable non-hematologic secondary cause (40, 41).

## Conclusions

From a clinical point of view, the main purpose of asthma treatment is to reduce symptoms and the recurrence of exacerbations. Mepolizumab proves its efficacy in the specific phenotype of asthmatic patients with intense eosinophilic airway inflammation and recurrent exacerbations. As often stated in children, we believe that FEV_1_ is not the most appropriate marker to detect mepolizumab's beneficial effects. Due to its capacity to target specific inflammatory type 2 mediators, mepolizumab represents a milestone in the application of personalized medicine. The prescription of mepolizumab for the treatment of severe asthma has expanded rapidly and this drug, currently used in adults, has also been registered for children, despite most of the scientific evidence in literature coming from adults. More data on efficacy in pediatric patients would be necessary to confirm its promising effects. Further clinical trials in the pediatric population are also important to prove long-term safety and the impact of this medication on the natural history of the disease. Finally, the identification of new biomarkers could be useful to predict real benefits from therapy with mepolizumab and to establish its optimal duration.

## Author Contributions

RC and NU discussed the writing project. RC, NU, FPe, and OF wrote the manuscript with significant support from FPo, EP, and AO. NU and RC supervised the final manuscript. All authors contributed to the article and approved the submitted version.

## Conflict of Interest

The authors declare that the research was conducted in the absence of any commercial or financial relationships that could be construed as a potential conflict of interest.

## Publisher's Note

All claims expressed in this article are solely those of the authors and do not necessarily represent those of their affiliated organizations, or those of the publisher, the editors and the reviewers. Any product that may be evaluated in this article, or claim that may be made by its manufacturer, is not guaranteed or endorsed by the publisher.
